# The Relationship between Lymphocyte Subsets and the Prognosis and Genomic Features of Lung Cancer: A Retrospective Study

**DOI:** 10.7150/ijms.56928

**Published:** 2021-03-25

**Authors:** Shuiping Dai, Pengwei Ren, Jing Ren, Lan Yang, Weimin Li

**Affiliations:** 1 Center of Gerontology and Geriatrics, West China Hospital, Sichuan University, Chengdu, China.; 2 Clinical Research Center for Respiratory Diseases, West China Hospital, Sichuan University, Chengdu, China.; 3 Nursing Department, West China Hospital, Sichuan University, Chengdu, China.; 4 Department of Respiratory and Critical Care Medicine, West China Hospital, Sichuan University, Chengdu, China.

**Keywords:** CD3+ T lymphocytes, CD8+ T lymphocytes, EGFR mutation, lung cancer, prognosis.

## Abstract

**Background:** It has been shown that the prognosis of malignant tumors was closely related to the composition and function of immune system, which was associated with genomic features. However, the prognostic value of peripheral T lymphocyte subsets and its relationship with genomic features in lung cancer has not been analyzed extensively. Therefore, this study was intended to evaluate the relationship between lymphocyte subsets and the prognosis and genomic features of lung cancer.

**Methods:** 598 lung cancer patients with complete data were included in this study between 2011 and 2018. Kaplan-Meier method and Pearson analyses were conducted to study the prognostic value of CD3+, CD4+, CD8+ T lymphocytes and the rate of CD4/CD8.

**Results:** Patients with *EGFR* mutation has lower mean percentage of CD8+ lymphocytes than patients with *EGFR* wild-type (24.71 versus 26.62, respectively, *P*=0.041). Patients with high CD3 had better OS than those with low (27 versus 14 months, *P*=0.002). Patients with higher CD4 and CD4/CD8 rate had longer OS than with lower (27 versus 12 months, *P*=0.002; 25 versus 9 months, *P*=0.008, respectively). Patients with high CD8 had poor PFS than low group (6 versus 11 months,* P*=0.009). There was a negative correlation between CD3+ and CD4+ cells and OS in smoking stage Ⅱ female lung cancer patients (PCC = 0.626, *P*<0.05; PCC = 0.534, *P*<0.05, respectively). In stage Ⅰ male lung cancer patients, CD8+T cell is negatively correlated with OS and PFS (PCC = 0.295, *P*<0.05; PCC = 0.280, *P*<0.05, respectively)

**Conclusions:** Lung cancer patients with *EGFR* mutation had lower percentage of CD8+ lymphocytes. Lymphocyte subsets might be potential prognostic biomarkers of lung cancer, but they are affected by gender and tumor stage.

## Background

Lung cancer is one of the most common malignant tumors with the highest mortality rate in the world 1. It accounts for about a quarter of all cancer deaths 2. Although the treatment of lung cancer, such as surgery, radiotherapy, chemotherapy and targeted therapy, has made great progress in recent years, the prognosis of lung cancer is still poor because most patients have been diagnosed in the locally advanced stage or in the stage of extensive metastasis 3. The five-year survival rate for patients with localized lung cancer is about 55%, while for patients with distant metastasis is only 4% 4, 5.

A series of studies have shown that the occurrence, development and prognosis of malignant tumors are closely related to the composition and function of immune system 6, 7. The systemic effects of tumor can make distant tissues and organs change in favor of metastasis and promote the progress of tumor 8-13. The metastasis of lung cancer is related to the increase of circulating tumor cells 14, which can be influenced by the change of CD3+, CD4+ cells in peripheral blood 15. A study about laryngeal squamous cell cancer indicates lower CD4/CD8 ratios are related to recurrent disease and a higher level of CD3 and CD4 is associated with nodal metastasis 16. The increased risk of lung cancer is associated with low CD4 cell count and low CD4/CD8 ratio 17. However, the prognostic value of peripheral T lymphocytes subsets in lung cancer has not been proved by studies with large data.

It has been reported that the genomic features of lung cancer were associated with inflammatory tumor-infiltrating lymphocytes 18. For example, the infiltrating B lymphocyte abundance was higher in the *EGFR*-mutated patients 19. However, the relationship between genomic features and circulating lymphocyte subsets was not clear. Hence, our study was aimed to evaluate the relationship between lymphocyte subsets and the prognosis and genomic features of lung cancer.

## Methods

### Patients and data collection

We retrospectively reviewed the medical records of patients who diagnosed with lung cancer at West China Hospital, Sichuan University, from January 2011 to January 2018. Inclusion criteria were: lung cancer patients, age > 18 years, with enough clinical and blood test data, no previous treatment for lung cancer before diagnosis. Criteria for exclusion from this study were: individuals with other malignancies or combined with immune system diseases, with an infection history or using drugs which could affect their immune system within one month before diagnosis.

Clinical and pathological data of included patients were collected through the medical record management system of West China Hospital. The basic information collected were: age at diagnosis, gender, smoking history, and histology. TNM stage of all patients were restaged according to the 8^th^ International Classification System for Lung Cancer 20. Peripheral indicators were obtained <1 week prior to the first treatment, including the proportion of CD3+, CD4+, CD8+ T lymphocyte subpopulations and the ratio of CD4/CD8.

### *EGFR* mutation, *ALK* and* ROS-1* rearrangements, and PD-L1 expression testing

Tumor specimens used for epidermal growth factor receptor (*EGFR*) mutation, anaplastic lymphoma kinase (*ALK*), proto-oncogene receptor tyrosine kinas (*ROS-1*) rearrangements, and programmed death-ligand 1 (PD-L1) expression detection were obtained from surgery, bronchoscopy, computed tomography (CT)-guided biopsy, and pleural effusion. *EGFR* mutation detection was carried out using Amplification Refractory Mutation System technology as the ADx EGFR Mutations Detection Kit (Amoy Diagnostics, China) had been approved for clinical application by the State Food and Drug Administration in China. *ALK* and *ROS-1* gene testing and PD-L1 expression was conducted on biopsy samples using immunostaining or the fluorescence *in situ* hybridization (FISH) method.

### Statistical analysis

The survival data included overall survival (OS) and progression-free survival (PFS). OS was defined as the time from the start of treatment to death from any cause. PFS was the time from start of treatment to radiographic or clinical progression or death from any cause. Patients who had not progressed or died at the last time of follow-up were censored at the date of the last contact.

X-tile software (Yale University, New Haven, CT, USA) was used to determine the optimal cut-off values of the proportion of CD3, CD4, CD8, CD4/CD8, neutrophil to lymphocyte ratio (NLR)and platelet to lymphocyte ratio (PLR) for predicting lung cancer prognosis^21^. Kaplan-Meier curves were used for the analysis of OS and PFS. The difference between groups were compared by the log-rank test. Canonical Correlation Analysis (Canoco 5.0) and Pearson analysis were used to evaluate the correlation between CD3, CD4, CD8, CD4/CD8, NLR, PLR, and other characteristics of lung cancer and PFS, OS. The comparation of lymphocyte subsets between different genomic features was performed by t-test. Two-tailed* P* values of <0.05 were considered to define statistical significance. All these statistical analyses were carried out using SPSS 20.0 (IBM Corp., Armonk, NY, USA).

## Results

### Patients characteristics

597 lung cancer patients with enough clinical and peripheral blood data were included in this study. The baseline characteristics of these patients were shown in Table 1. Median age of these patients was 61 years old. More than half of patients were male (58.96%) and smokers (53.77%). 397 (66.5%) individuals were diagnosed with adenocarcinoma. The majority of patients were diagnosed at late tumor stage (Ⅲ-Ⅳ, 72.53%). The prevalence of *EGFR* mutation in tumor cell was 45.55%, *ALK* rearrangement 8.11%, *ROS-1* rearrangement 2.56%, PD-L1 expression positive 59.01%.

The percentage of T-lymphocyte subsets in lung cancer patients with different genomic aberrations and PD-L1 expression was shown in Table 2. Patients with *EGFR* mutation has lower percentage of CD8+ lymphocytes than patients with *EGFR* wild-type (24.71±8.69 and 26.62±9.23, respectively, *P*=0.041). There was no significant difference in the proportion of T-lymphocyte subsets between PD-L1 expression positive and negative patients.

### Survival outcomes

The mean percentage of pretreatment circulating CD3+, CD4+, CD8+ lymphocytes and CD4/CD8 ratio were 66.69%, 36.98%, 25.27%, and 1.76 respectively. Median NLR was 3.39 and PLR was 138.94. The cut-off values of these indicators for predicting prognosis in lung cancer patients were defined by X-tile software. CD3 was 67.25%, CD4 was 42%, CD8 was 33.9%, CD4/CD8 was 1.085, NLR was 1.9, and PLR was 213.8. Patients were stratified into 2 groups (low group and high group) by the cut-off value of these indicators, when Kaplan-Meier curves and log-rank test were used for the analysis of OS and PFS.

**Figure 1:**
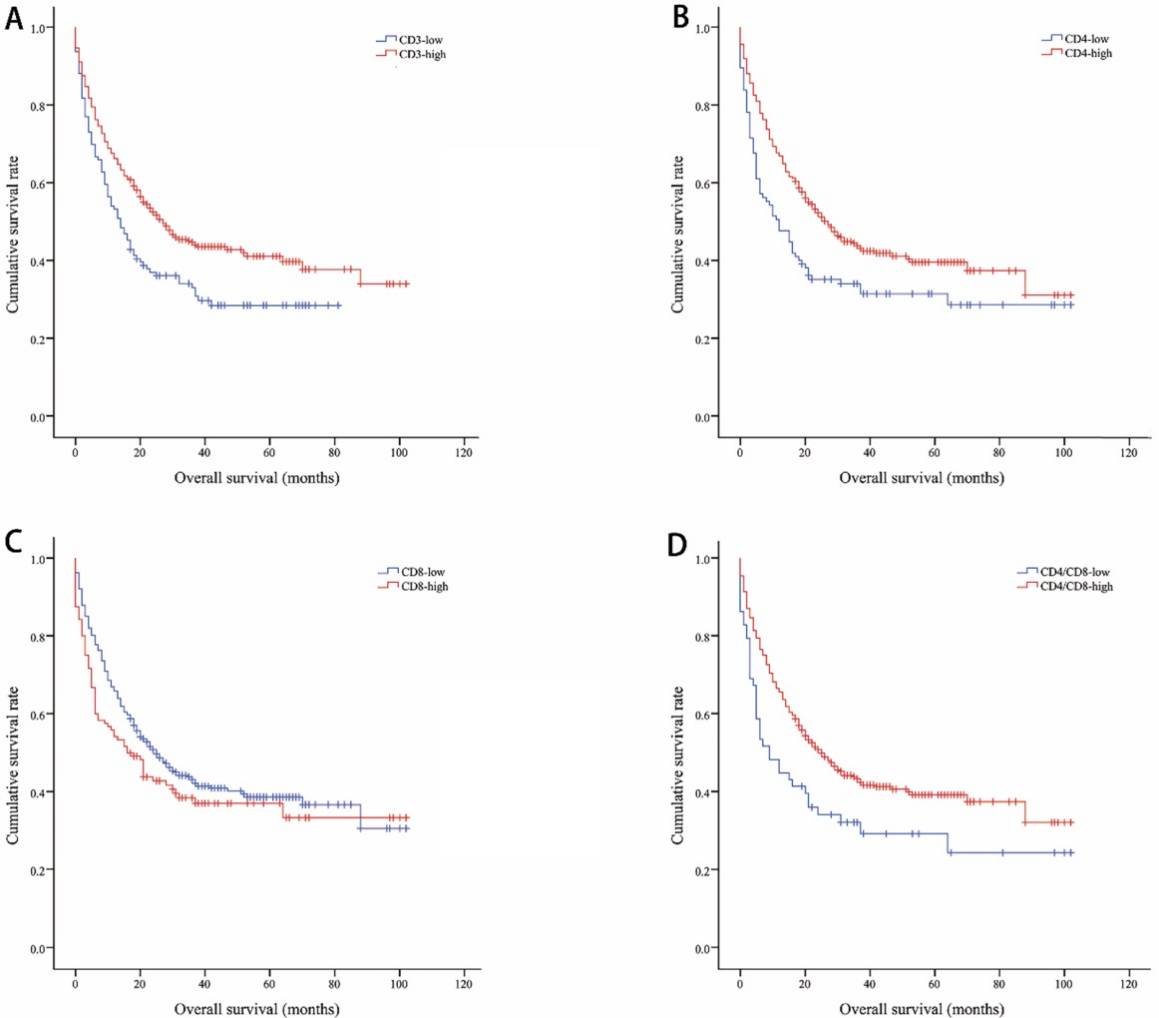
Kaplan-Meier curves showing overall survival by CD3 (A), CD4 (B), CD8 (C), CD4/CD8 (D)

**Figure 2:**
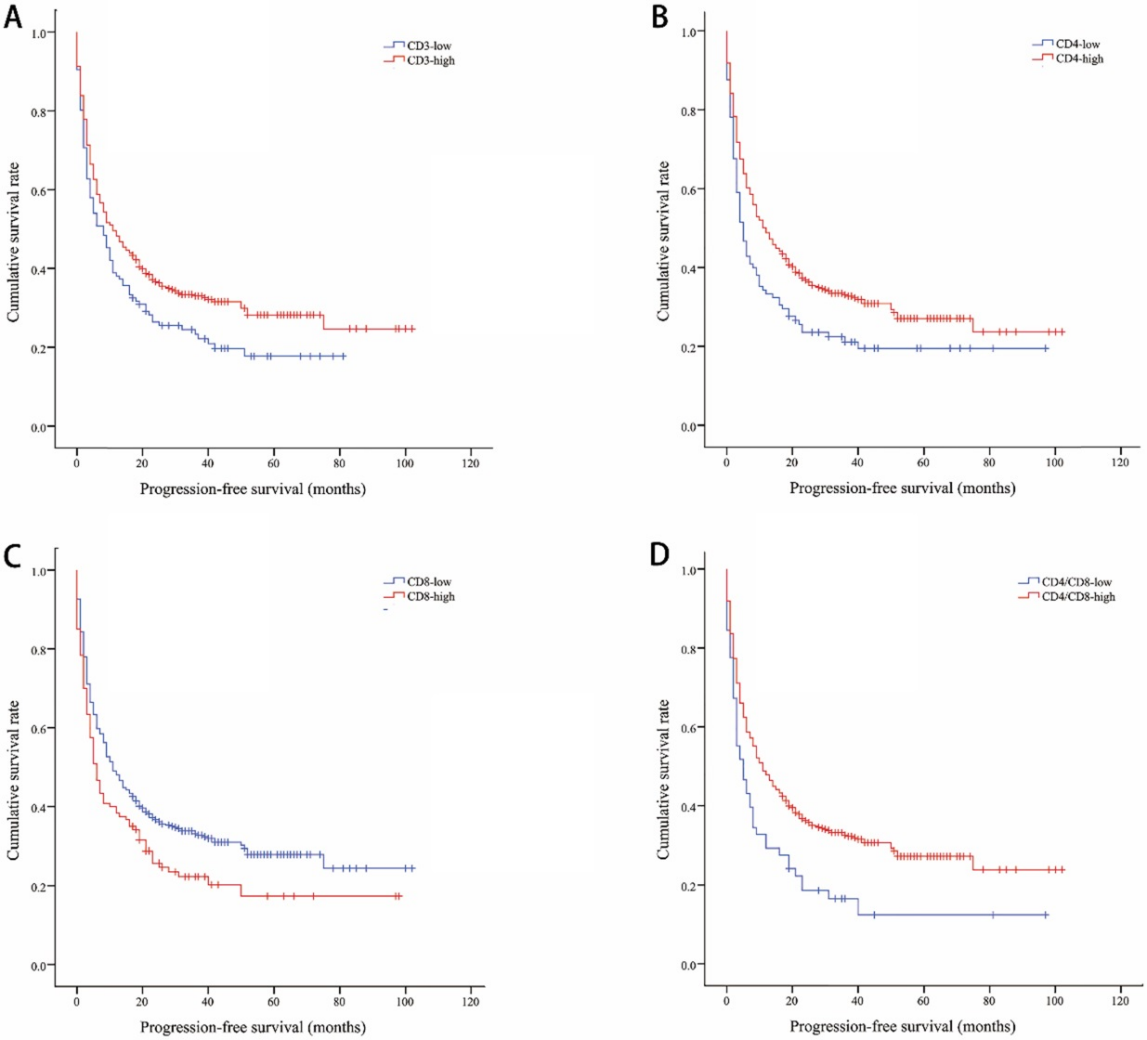
Kaplan-Meier curves showing progression-free survival by CD3 (A), CD4 (B), CD8 (C), CD4/CD8 (D)

The median OS of all included lung cancer patients was 23 months (95% confidence interval (CI), 18.56-27.44), and the median PFS was 10 months (95%, 7.76-12.24). The OS rates of patients according to CD3, CD4, CD8, and CD4/CD8 were shown in Figure 1. The CD3-high group had higher OS than the CD3-low group, with a median OS of 27 versus 14 months (*P*=0.002). Patients with higher CD4 shown longer OS (CD4-high versus CD4-low, 27 versus 12 months, *P*=0.002). The higher rate of CD4/CD8 also had better OS (CD4/CD8-high versus CD4/CD8-low, 25 versus 9 months, *P*=0.008). However, OS showed no significant difference between CD8-high and CD8-low group (16 versus 25 months, *P*=0.1). Patients with higher CD3, CD4, or CD4/CD8 rate had longer PFS (11 versus 8 months, *P*=0.017; 11 versus 5 months, *P*=0.003; 11 versus 5 months, *P*=0.002, respectively). Conversely, the CD8-high group had poor PFS than the CD8-low group (6 versus 11 months, *P*=0.009). The PFS rates of patients according to CD3, CD4, CD8, and CD4/CD8 were shown in Figure 2. The OS and PFS was significant longer in the NLR-low group (OS, 88 versus 19 months, *P*<0.001; PFS, 26 versus 7 months, *P*<0.001, respectively), which was also found in the PLR-low group (OS, 29 versus 11 months, *P*<0.001; PFS, 13 versus 4 months, *P*<0.001, respectively) (Figure S1).

### The correlation between lymphocyte subsets and prognosis

**Figure 3:**
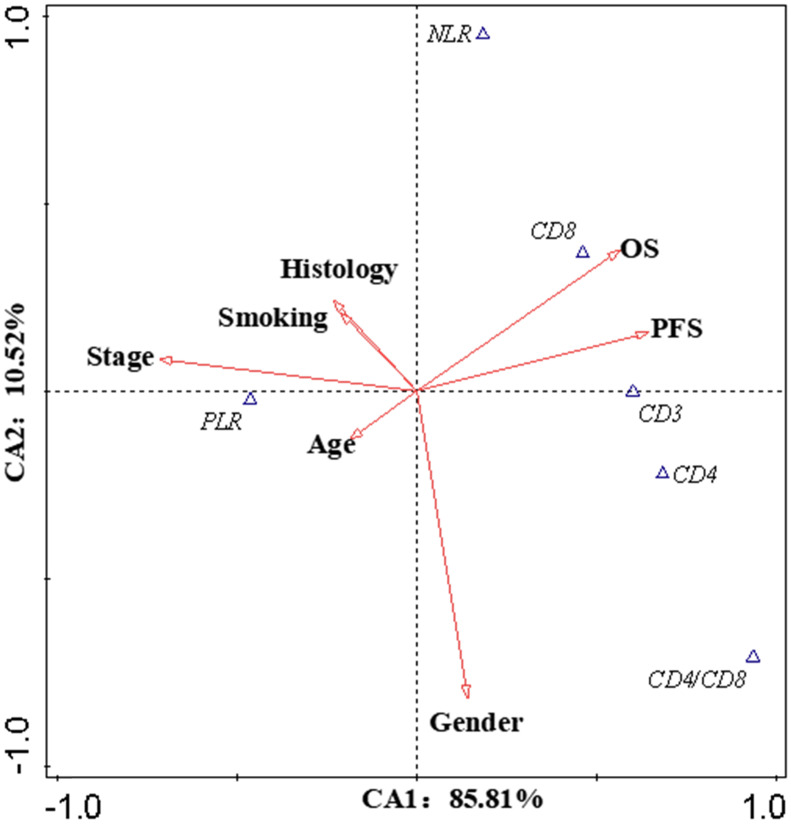
Canonical Correlation Analysis. The canonical correlation analysis (CCA) were performed in this work to further elucidate the interactions among the prognostic biomarkers and the potential impact factors (IF). The values on the axes indicate adjusted explained variations. Triangles mean prognostic biomarkers, arrows mean IF (except OS and PFS). Stage and gender were the dominant IF, which contributed to the variations in CA1 and CA2, respectively. According to the intersection angle between the biomarkers and the IF, CD8 and PLR might be the candidates to estimate the prognosis.

**Figure 4:**
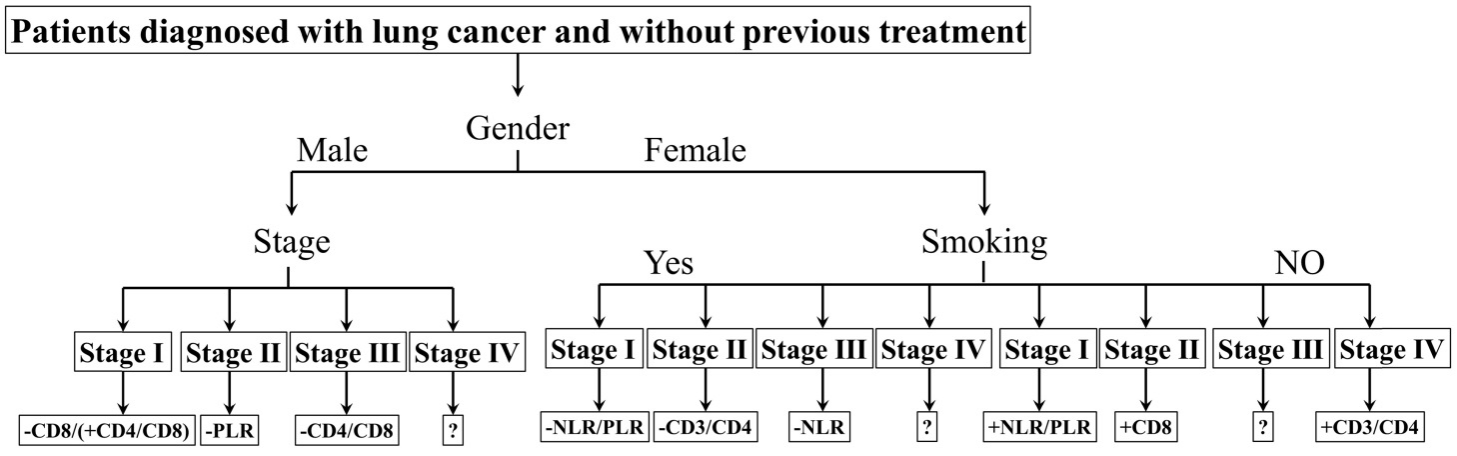
The biomarkers to assess the prognosis. +: positive correlation; -: negative correlation; ?: no appropriate biomarkers in this work. Of male patients, in stage Ⅰ, there was a negative correlation between CD8 and prognosis, a positive correlation between CD4/CD8 and prognosis; in stage Ⅱ, a negative correlation between PLR and prognosis; in stage Ⅲ, a negative correlation between CD4/CD8 and prognosis; in stage Ⅳ, no appropriate biomarker was found. NLR/PLR was negatively correlated with prognosis in smoking stage Ⅰ female patients, while positively in no-smoking stage Ⅰ female patients. CD4/CD8 was negatively correlated with prognosis in smoking stage Ⅱ female patients, while positively in no-smoking stage Ⅳ female patients. There was a negative correlation between NLR and prognosis in smoking stage Ⅲ female patients; a positive correlation between CD8 and prognosis in no-smoking stage Ⅱ female patients. No appropriate biomarker was found in smoking stage Ⅳ and no-smoking stage Ⅲ female patients.

Canonical Correlation Analysis was used to analysis the correlations between lymphocyte subsets, clinical characteristics and prognosis of lung cancer. Four axes were determined *via* CCA, and the cumulative fitted variations explained by CA1 (85.81%) and CA2 (10.52%) were up to 96.33%, indicating the validity of this analysis. As shown in Figure 3, gender and stage had potential impacts on PFS and OS. Smoking had an impact on the prognosis of female patients. According to clinical characteristic, the patients were divided into different groups to explore the relationship between lymphocyte and prognosis. In non-smoking stage Ⅱ female lung cancer patients, CD8+T cell was positively correlated with OS and PFS (Pearson correlation coefficient (PCC) = 0.587, *P*<0.05; PCC = 0.586, *P*<0.05, respectively) (Figure S2). There was a negative correlation between CD3+ and CD4+ cells and OS in smoking stage Ⅱ female lung cancer patients (PCC = 0.626, P<0.05; PCC = 0.534, P<0.05, respectively) (Figure S3). In stage Ⅰ male lung cancer patients, CD8+T cell was negatively correlated with OS and PFS (PCC = 0.295, P<0.05; PCC = 0.280, P<0.05, respectively) (Figure S4). Indicators related to the prognosis of patients with lung cancer were listed in Figure 4.

## Discussion

This study indicated that the high percentages of peripheral CD3+, CD4+ T cells and CD4/CD8 ratio were associated with longer OS in lung cancer patients. However, the high baseline proportion of CD8+ T cells was significant predictors of poor PFS. Lung cancer patients with *EGFR* mutation had lower percentage of CD8+ lymphocytes.

Previous study observed that the risk of lung cancer was associated with low CD4 cell count and low CD4/CD8 ratio 17. Peripheral CD8+T cells was higher in lung cancer patients compared to the health, while CD3+ and CD4+T cells, and CD4/CD8 ratio was significantly lower 22. The relationship of lower CD4+/CD8+ ratios and recurrent diseases and higher level of CD3+ and CD4+ cells and nodal metastasis was found in laryngeal squamous cell cancer^16^.

In our study, the prevalence of *EGFR* mutation in tumor cells was 45.55%, *ALK* rearrangement 8.11%, *ROS-1* rearrangement 2.56%, which was similar to the results of other studies 23-25. The expression of PD-L1 in tumor cells was consistent with previous studies 26, 27. We found patients with *EGFR* mutation had lower percentage of circulating CD8+ T cells. A precious study found there was no significant difference in the proportion of tumor infiltration CD8+ T cells between *EGFR* mutation and *EGFR* wild-type patients 28. Future researches were need to clarify the relationship of T lymphocytes and *EGFR* mutation in lung cancer.

The survival curve of this study showed that peripheral CD4+ T cells but not CD8+ T cells were associated with OS in lung cancer. A previous research also indicated that tumor infiltrating CD4+ T cells, not CD8+ T cells, were associated with favorable prognosis in non-small cell lung cancer 29. In contrast, an meta-analysis found that CD4+ T cells in tumor compartment had no influence on OS, while CD8+ T cells were associated with better prognosis in terms of OS in non-small cell lung cancer patients 30. They also found that elevated CD3+ T cells was associated with improved OS, which was constant with our results. Another meta-analysis clarified that high level of CD3+, CD4+, and CD8+ T cells infiltration showed better OS in lung cancer patients 31. The distant prognosis value of T cells in different sites was needed to be explored by more researches. Increased CD8+CD28+ T cells independently predicted favorable OS and PFS in lung adenocarcinoma, while high levels of CD8+CD28- T cells predicted unfavorable OS and PFS in lung squamous cell carcinoma 32. The prognosis value was associated with the subtypes of CD8+ T cells. Therefore, the prognostic value of different subtypes of CD4+ and CD8+ T cells in lung cancer were needed to be studied by more researches.

## Conclusion

Lung cancer patients with *EGFR* mutation had lower percentage of CD8+ lymphocytes. High level of peripheral CD3+ T cells predict longer OS in lung cancer patients, while high level of peripheral CD8+ T cells are associated with poor PFS. Lymphocyte subsets might be potential prognostic biomarkers of lung cancer, but they were affected by gender and tumor stage.

## Supplementary Material

Supplementary figures and tables.

## Figures and Tables

**Table 1:** The clinical-pathological characteristics of patients (N (%)).

Age, years	Median	61
	Range	20-87
Gender	Male	352 (58.96)
	Female	245 (41.04)
Smoking	No	276 (46.23)
	Yes	321 (53.77)
Histology	Adenocarcinoma	397 (66.50)
	Squamous carcinoma	100 (16.75)
	Others	100 (16.75)
Stage	Ⅰ	103 (17.25)
	Ⅱ	61 (10.22)
	Ⅲ	167 (27.97)
	Ⅳ	266 (44.56)
CD3 (%)	Mean	66.69
	Standard deviation	10.89
CD4 (%)	Mean	36.98
	Standard deviation	9.44
CD8 (%)	Mean	25.27
	Standard deviation	9.19
CD4/CD8	Mean	1.76
	Standard deviation	1.53
*EGFR* mutation	Positive	169 (45.55%)
	Negative	202 (54.45%)
*ALK* rearrangement	Positive	36 (8.11%)
	Negative	408 (91.89%)
*ROS-1* rearrangement	Positive	9 (2.56%)
	Negative	343 (97.44%)
PD-L1 expression	Positive	95 (59.01%)
	Negative	66 (40.99%)

**Table 2:** Circulating T-lymphocyte subsets of different patients with lung cancer (mean (±standard deviation)).

	CD3 (%)	*P*-value	CD4 (%)	*P*-value	CD8 (%)	*P*-value	CD4/CD8	*P*-value
EGFR mutation		0.368		0.389		0.041		0.938
Positive	67.26 (±10.90)		37.72 (±8.77)		24.71 (±8.69)		1.80 (±0.98)	
Negative	68.25 (±10.28)		36.92 (±9.11)		26.62 (±9.23)		1.78 (±2.59)	
ALK rearrangement		0.774		0.231		0.184		0.289
Positive	67.99 (±9.18)		35.82 (±8.95)		27.19 (±8.08)		1.48 (±0.74)	
Negative	66.46 (±10.57)		37.69 (±8.98)		25.18 (±8.74)		1.82 (±1.93)	
ROS-1 rearrangement		0.768		0.177		0.334		0.816
Positive	69.02 (±11.85)		41.90 (±8.33)		22.64 (±5.95)		1.96 (±0.65)	
Negative	67.96 (±10.58)		37.78 (±9.03)		25.49 (±8.77)		1.80 (±2.04)	
PD-L1 expression		0.834		0.308		0.075		0.069
Positive	69.58 (±9.29)		37.70 (±9.75)		27.50 (±10.46)		1.58 (±0.722)	
Negative	69.94 (±11.72)		39.19 (±8.64)		24.79 (±8.68)		1.80 (±0.792)	

## Abbreviations

EGFR: Epidermal growth factor receptor
OS: Overall survival
PFS: Progression-free survival
ROS-1: Proto-oncogene receptor tyrosine kinase
ALK: Anaplastic lymphoma kinase
PD-L1: Programmed death-ligand 1
HR: Hazard ratio
CI: Confidence interval
PCC: Pearson correlation coefficient
