# Supplementary material for: The Relationship between Lymphocyte Subsets and the Prognosis and Genomic Features of Lung Cancer: A Retrospective Study
Source: Int J Med Sci. 2021 Mar 25;18(10):2228–34. doi: 10.7150/ijms.56928 (PMC8040422; doi:10.7150/ijms.56928)
Supplement: Supplementary file 1 — Supplementary figures and tables. [file ijmsv18p2228s1.pdf]

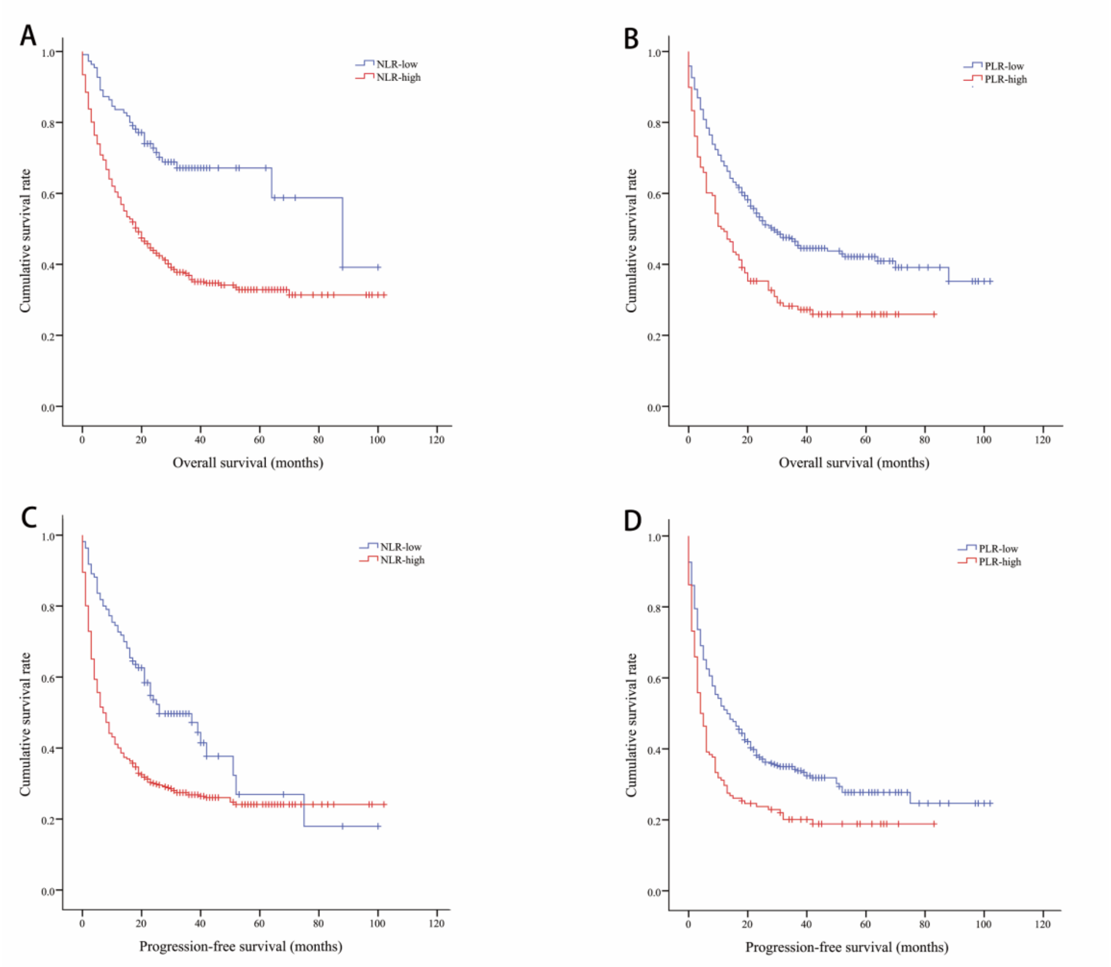

Figure S1 Kaplan-Meier curves showing overall survival and progression-free survival by NLR and PLR. Curves showed overall survival by NLR (A), PLR (B). Curves showed progression survival by NLR (C), PLR (D).

NLR, neutrophil-to-lymphocyte ratio; PLR, platelet-to-lymphocyte ratio.

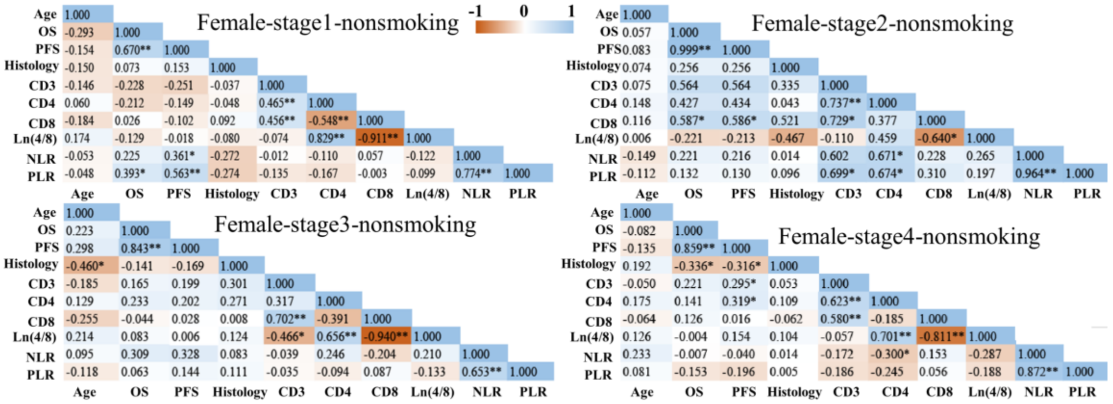

Figure S2 The correlation between indicators and prognosis in nonsmoking female lung cancer patients

**: There was a significant correlation at the 0.01 level (bilateral); *: There was a significant correlation at the 0.05 level (bilateral).

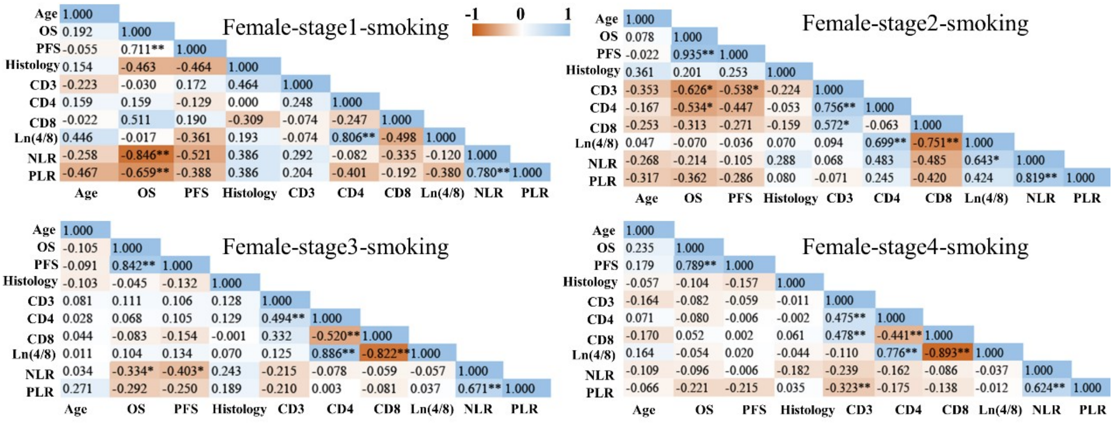

Figure S3 The correlation between indicators and prognosis in smoking female lung cancer patients

**: There was a significant correlation at the 0.01 level (bilateral); *: There was a significant correlation at the 0.05 level (bilateral).

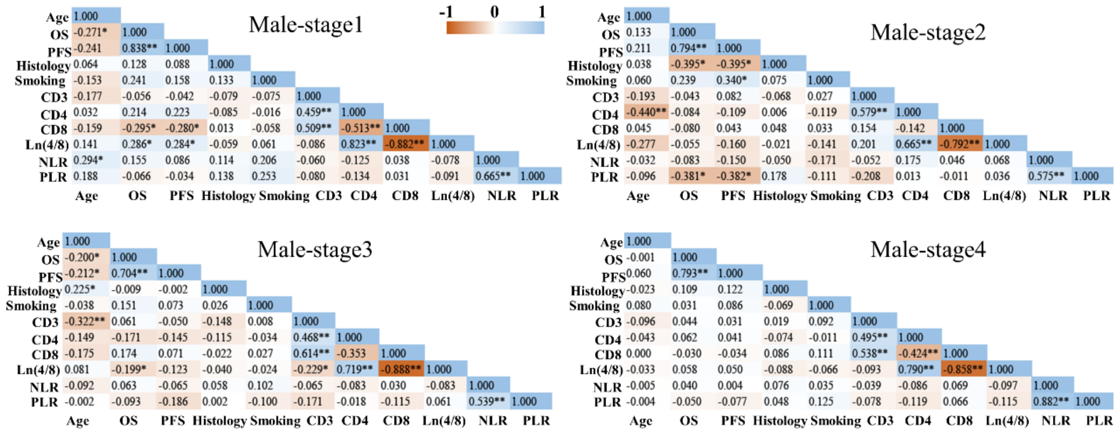

Figure S4 The correlation between indicators and prognosis in male lung cancer patients

**: There was a significant correlation at the 0.01 level (bilateral); *: There was a significant correlation at the 0.05 level (bilateral).
